# The POP (Permanent Supportive Housing Overdose Prevention) Study: protocol for a hybrid type 3 stepped-wedge cluster randomized controlled trial

**DOI:** 10.1186/s13012-023-01278-z

**Published:** 2023-06-07

**Authors:** Kelly M. Doran, Allison Torsiglieri, Stephanie Blaufarb, Patricia Hernandez, Emily Melnick, Lauren Velez, Charles M. Cleland, Charles Neighbors, Megan A. O’Grady, Donna Shelley

**Affiliations:** 1 Department of Emergency Medicine, NYU School of Medicine, New York, NY USA; 2 Department of Population Health, NYU School of Medicine, New York, NY USA; 3 Metro Team, Corporation for Supportive Housing, New York, NY USA; 4 Department of Public Health Sciences, University of Connecticut School of Medicine, Farmington, CT USA; 5 Department of Public Health Policy and Management, NYU School of Global Public Health, New York, NY USA; 6 Global Center for Implementation Science and Practice, NYU School of Global Public Health, New York, NY USA

**Keywords:** Overdose, Stepped-wedge cluster randomized controlled trial, Implementation study, Housing, Protocol

## Abstract

**Background:**

Permanent supportive housing (PSH)—subsidized housing paired with support services such as case management—is a key part of national strategic plans to end homelessness. PSH tenants face high overdose risk due to a confluence of individual and environmental risk factors, yet little research has examined overdose prevention in PSH.

**Methods:**

We describe the protocol for a hybrid type 3 stepped-wedge cluster randomized controlled trial (RCT) of overdose prevention practice implementation in PSH. We adapted evidence-based overdose prevention practices and implementation strategies for PSH using input from stakeholder focus groups. The trial will include 20 PSH buildings (with building size ranging from 20 to over 150 tenants) across New York City and New York’s Capital Region. Buildings will be randomized to one of four 6-month intervention waves during which they will receive a package of implementation support including training in using a PSH Overdose Prevention (POP) Toolkit, time-limited practice facilitation, and learning collaboratives delivered to staff and tenant implementation champions appointed by each building. The primary outcome is building-level fidelity to a defined list of overdose prevention practices. Secondary and exploratory implementation and effectiveness outcomes will be examined using PSH staff and tenant survey questionnaires, and analysis of tenant Medicaid data. We will explore factors related to implementation success, including barriers and facilitators, using qualitative interviews with key stakeholders. The project is being conducted through an academic-community partnership, and an Advisory Board including PSH tenants and other key stakeholders will be engaged in all stages of the project.

**Discussion:**

We describe the protocol for a hybrid type 3 stepped-wedge cluster RCT of overdose prevention practice implementation in PSH. This study will be the first controlled trial of overdose prevention implementation in PSH settings. The research will make a significant impact by testing and informing future implementation strategies to prevent overdose for a population at particularly high risk for overdose mortality. Findings from this PSH-focused research are expected to be broadly applicable to other housing settings and settings serving people experiencing homelessness.

**Trial registration:**

ClinicalTrials.gov, NCT05786222, registered 27 March 2023.

**Supplementary Information:**

The online version contains supplementary material available at 10.1186/s13012-023-01278-z.

Contributions to the literature

• Permanent supportive housing (PSH) is an evidence-based intervention for homelessness and is expanding across the USA. Overdose is a critical issue in PSH, yet overdose prevention in this setting has been sparsely researched.
• This manuscript describes the protocol for a stepped-wedge cluster randomized controlled trial of overdose prevention practice implementation in PSH. This trial will be the largest study of overdose prevention implementation in PSH to date.
• The study will test an implementation framework and novel implementation strategies in PSH, with potential broad relevance to other housing settings.

## Background

The overdose crisis continues to worsen in the USA, and health disparities related to overdose have widened in recent years [1–4]. Amidst these widening disparities, the connections between social determinants of health and overdose have become increasingly clear. For example, research has shown that homelessness is strongly associated with heightened risk for overdose [5–8].

Permanent supportive housing (PSH)—subsidized housing paired with support services such as case management—is a key part of national strategic plans to end homelessness [9]. PSH is generally targeted to people who have been chronically homeless and have significant health conditions, including substance use disorders and mental illness. Decades of evidence, including multiple RCTs, have proven that PSH is highly effective in durably resolving an individual’s homelessness, including for people with serious mental illness and people who use drugs [10–14]. There are currently 387,305 units of PSH across the USA, a number that has grown consistently in the past 15 years and that continues to grow [9, 15]. PSH is sometimes referred to as “Housing First,” which originated as a specific model, but now is a term often used more generally to describe the approach of placing people into housing without stepwise requirements or prerequisites of sobriety or “stability” of mental illness [16, 17]. The original Housing First model has harm reduction as a core tenet, but currently there is variability regarding the extent to which different PSH agencies embrace harm reduction [18].

While housing is critical to ending homelessness, placement into housing alone may not reduce overdose risk [10]. In fact, emerging evidence suggests relatively high overdose rates in PSH and similar housing settings such as single room occupancies (SROs), likely due to a confluence of individual and environmental risk factors [19–21]. For example, in a 2019 survey of leaders from 49 New York PSH agencies, 63% of agencies reported at least one opioid-involved overdose among their PSH tenants in the past year, and at least 118 tenants were known to have died from an overdose in the past 5 years [20]. PSH leaders completing the survey also identified multiple modifiable gaps related to overdose prevention in PSH [20].

Evidence-based practices for overdose prevention exist but their implementation in PSH has been sparsely studied. In general, integration of harm reduction principles and substance use-related initiatives in PSH settings is variable [22–25]. Limited non-experimental research has examined implementation of “Housing First” principles including harm reduction into PSH [26]. One study suggested that training and practice facilitation positively impacted PSH staff knowledge and attitudes toward harm reduction, and was met with high satisfaction [26]. Researchers in Vancouver, Canada, conducted qualitative research examining overdose risk and selected overdose prevention interventions (overdose response buttons and peer-led naloxone training and distribution) in PSH and SROs [21, 27, 28]. However, overall there is a paucity of high-quality evidence related to harm reduction in PSH [29] and there remains a significant gap in the evidence related to the effective implementation of overdose prevention practices in these settings. Indeed, to our knowledge, there has not yet been any experimental research examining implementation of overdose prevention practices in PSH. We seek to fill this gap by conducting a stepped-wedge cluster RCT to study the implementation of overdose prevention practices across 20 PSH buildings in New York.

## Methods

### Study design

This study is a hybrid type 3 trial using a stepped-wedge cluster RCT design [30], with the primary goal of studying building-level implementation of overdose prevention practices in PSH and a secondary goal of examining effectiveness on clinically relevant tenant outcomes. A stepped-wedge cluster RCT design was chosen because it can provide rigorous evidence for intervention effects while allowing all participating buildings to eventually receive the intervention, in contrast to traditional two-arm RCTs. The stepped-wedge design also presents advantages including feasibility related to staggered intervention start times, and improved power versus parallel cluster RCTs.

The study is funded by the National Institute on Drug Abuse of the National Institutes of Health and was approved by the Institutional Review Board at NYU Grossman School of Medicine. The study’s external Data Safety and Monitoring Board meets twice yearly. This manuscript adheres to CONSORT reporting guidelines for cluster randomized trials (checklist provided as an Additional file 1: Table S1).

### Conceptual frameworks

We drew on the EPIS (Exploration, Preparation, Implementation, Sustainment) framework to guide the study’s design [31, 32]. This multilevel framework includes both process (“phases”) and determinant (“constructs”) components. We previously completed the Exploration phase through surveys conducted with PSH building leaders in New York to identify the scope of overdose and related gaps in PSH [20]. The Preparation phase of our work uses key stakeholder focus groups to refine a package of overdose prevention practices and implementation strategies for PSH. The stepped-wedge cluster RCT encompasses both the implementation and sustainment phases of EPIS, as further described in the sections that follow.

We consider key drivers of overdose risk and overdose prevention practice implementation in PSH within the EPIS constructs of outer context, inner context, bridging factors, and innovation factors. We organize these further within Rhodes’ Risk Environment Framework, which categorizes physical, social, economic, and policy-level risks at the level of the micro- and macro-environment [33]. Researchers have previously used Rhodes’ Risk Environment Framework to categorize environmental factors that confer risk for overdose in SROs, such as lack of shared spaces and rules or norms which may lead to using drugs alone [21, 34, 35]; many of these risks are common to PSH settings. We hypothesize that building receipt of an intervention package that considers the inner and outer context in PSH—as we are developing in the study’s Preparation phase—will improve fit and, subsequently, the likelihood that buildings will successfully implement the overdose prevention practices.

The overall project is grounded in the philosophies of community based participatory research (CBPR) [36]. From the beginning of the project, academic investigators have worked in partnership with the Metro Team (covering New York, New Jersey, and Pennsylvania) of the Corporation for Supportive Housing (CSH). CSH is intimately familiar with strengths, assets, and barriers specific to PSH. In the study’s Preparation phase, the planned overdose prevention practices and implementation strategy package are being refined based on input from PSH leaders, frontline staff, and tenants. Additionally, the project is guided by a Study Advisory Board (SAB) at all stages of the research. The SAB includes PSH tenants and staff, people with lived experience of drug use, and representatives from relevant community (e.g., harm reduction, housing advocacy) and governmental (e.g., city and state departments of health) organizations.

### Setting

The stepped-wedge cluster RCT will include 20 PSH buildings in New York City (NYC) and New York’s Capital Region. These areas were chosen to provide a diversity of settings, enhancing generalizability. New York’s Capital Region encompasses the mid-sized metropolitan areas of Albany, Troy, and Schenectady. This trial includes *congregate* PSH buildings, where PSH tenants live together and have access to onsite social services such as case management. In general, onsite services are provided by a nonprofit agency whose portfolio might include multiple PSH buildings as well as other homeless services. Separate property/building management companies may operate the physical housing/apartment complex. Some congregate PSH buildings are mixed-use buildings, also having affordable or market-rate units for non-PSH tenants. Congregate PSH stands in contrast to “scattered site” PSH, where PSH tenants live “scattered” in subsidized market units across the community. Our trial focuses on congregate PSH because this form of PSH is most amenable to building-level interventions and most generalizable to other types of buildings serving populations at high risk for overdose.

### Study population and eligibility

Twenty PSH buildings in NYC and New York’s Capital Region were selected for participation in the trial. CSH’s Metro Team conducted initial outreach to 57 nonprofit agencies in NYC and the Capital Region that provide PSH services, representing the large majority of such agencies in these areas. Agency leaders were provided with written information about the project. They were also invited to an information session held by Zoom and engaged in individual meetings to discuss the project. Agencies were asked to complete online building assessment questionnaires including questions about the size of the building, population served, number of fatal and nonfatal overdoses in the past year, and existing building practices related to overdose prevention. Questionnaires were completed by 25 agencies, encompassing 44 unique buildings. Of the 32 agencies to whom outreach was conducted who did not ultimately complete a questionnaire, 15 did not reply to outreach e-mails, 8 expressed that they were not interested in the project, and 9 were determined not to be eligible (e.g., due to not providing congregate PSH).

The CSH Metro Team and academic study team used the information gathered from their meetings with buildings and from the building assessment questionnaires to select buildings for participation. The primary selection criteria were that buildings had to have at least 20 PSH tenants and overdose had to be a significant concern, as demonstrated either by report of past overdoses on building assessment questionnaires or report from building leaders during meetings. Of the 44 buildings completing building assessment questionnaires, 2 were not eligible based on size and 2 expressed that they did not want to participate. From the remaining 40 buildings, we selected and invited 20 to participate; 3 buildings (from 2 agencies) declined and were replaced to reach 20 participating buildings. Selection prioritized buildings with high levels of overdose concern. Buildings also had to have some degree of “room to improve” in overdose prevention, as demonstrated by their building assessment questionnaire responses related to existing overdose prevention efforts or based on gaps in overdose prevention identified in meetings. To maximize generalizability, buildings were not required to demonstrate a certain level of readiness for implementing new overdose prevention practices; however, buildings did have to express willingness to engage in activities to support them in implementing new overdose prevention practices (e.g., practice facilitation meetings), and to participate in the research study procedures. We also aimed to ensure diversity in participating buildings (e.g., in population served or agency size). No more than two buildings were selected from any given nonprofit agency and, in the case of multiple buildings from the same agency, buildings were required to be geographically dispersed, have different directors, and operate independently, to avoid concerns related to “clustering.” Larger buildings were prioritized for selection in NYC given that Capital Region buildings tend to be smaller (see “Sample size” and “Power” sections).

Leaders from the 20 buildings participating in the trial completed a project participation agreement with CSH and a data sharing agreement with the academic study team.

### Intervention overview

The intervention is provision of multi-faceted implementation support to PSH buildings, with the goal of helping them implement a set of evidence-based practices to reduce tenant overdose. As detailed below, the intervention will be delivered by CSH. The implementation strategies to be tested and overdose prevention practices to be implemented and are being refined in a 1-year preparation phase. In this phase, insight to and feedback on the implementation strategies and overdose prevention practices are being gathered from multiple sources including: focus groups with PSH tenants, staff, and leaders in NYC and New York’s Capital Region; additional discussions with PSH staff and leaders from buildings participating in the study; Study Advisory Board meetings; and meetings with and review by content experts and other relevant stakeholders. In future publications we will present focus group results and the final implementation strategy and overdose prevention practice packages. In the sections that follow, we provide an overview of the anticipated implementation strategies to be tested and overdose prevention practices to be implemented.

### Implementation strategies to be tested

Each building participating in the trial will receive 6 months of support in implementing the overdose prevention practices. CSH will provide implementation support consisting of four primary strategies (see Table 1): (1) a POP (PSH Overdose Prevention) Toolkit and associated trainings, (2) tenant and staff implementation champions, (3) limited practice facilitation, and (4) learning collaboratives. These strategies—which are included in the compilation of implementation strategies from the Expert Recommendations for Implementing Change (ERIC) project [37]—were selected based on conversations between the academic study team and CSH about which implementation strategies were most likely to be accepted by and useful to PSH buildings. Together, we are calling this package of implementation support “technical assistance,” to align with language more commonly used by PSH buildings.

**Table 1:** Implementation strategy package

Strategy	Details
PSH Overdose Prevention (POP) Toolkit	- Detailed implementation manual/blueprint for the overdose prevention practices - Will be introduced to buildings in 3 pre-recorded seminars (≈ 90 min each) - Will include planning worksheets to be used in practice facilitation sessions and educational materials related to each overdose prevention practice
Tenant and staff implementation champions	- Implementation champions “dedicate themselves to supporting, marketing, and driving through an implementation, overcoming indifference or resistance” [37] - One staff member and two tenants will be appointed as implementation champions in each building. Tenant champions will receive an honorarium - Champions will work with the practice facilitator and other building stakeholders to implement and sustain overdose prevention practices in the building
Practice facilitation	- A staff member from CSH will be trained as a practice facilitator - The practice facilitator will work with tenant and staff champions, guiding and building their capacity to implement the overdose prevention practices - Initial practice facilitation meetings: tailored support in 3 workshops (≈ 90 min each) with champions and other key building staff/leaders. Meetings will focus on key questions and setting-specific problem solving - Ongoing coaching: after initial meetings, practice facilitator will lead biweekly coaching sessions for 3 months and will remain available for technical support and less frequent check-ins for up to 3 additional months - Practice facilitation sessions will be held remotely (e.g., Zoom)
Learning collaboratives	- 5 PSH buildings in the same intervention wave will form a learning collaborative - Monthly learning collaborative meetings for 6 months, facilitated by the practice facilitator. Meetings will be attended by implementation champions and, optionally, up to 1–2 other key staff/leaders from each building - Webinar format (e.g., Zoom) with discussion of ideas, challenges, and solutions related to implementing and sustaining overdose prevention practices

*Legend*: Planned strategies for supporting PSH buildings in implementing overdose prevention practices. The full implementation strategy package will be refined and finalized during the preparation phase of the study. CSH will administer the implementation support (also being called “technical assistance,” as this term is more commonly used by supportive housing agencies) to each building during its randomly assigned 6-month intervention period

Trainings, initial practice facilitation meetings and coaching sessions, and learning collaboratives are expected to amount to approximately 20 h over the first 3 months of each 6-month technical assistance period. More limited practice facilitation will continue in the next 3 months, in addition to ongoing learning collaborative meetings. Outside of scheduled meetings, building leaders and staff and tenant implementation champions are expected to spend additional time working to implement the overdose prevention practices in their building. Overall, the amount of time for active implementation activities is moderate, and deliberately designed to maximize generalizability in PSH settings given real-world resource constraints.

#### Overdose prevention practices to be implemented

Buildings will receive support for implementing overdose prevention practices in three core categories: (1) overdose response; (2) harm reduction; and (3) support for substance use disorder (SUD) treatment. While the exact list of specific overdose prevention practices within each category is being refined in the study’s preparation phase, examples of anticipated practices are shown in Table 2.

**Table 2:** Overdose prevention practices

Category	Overdose prevention practice examples
Overdose response	Create building-specific written overdose prevention and response plan; conduct formal debriefings after any overdose; provide support to staff and tenants related to trauma resulting from overdose; make naloxone readily available in the building; provide all tenants and staff with a naloxone kit and training on naloxone use and overdose response; systematically track information on overdoses in the building
Harm reduction	Uphold protocols that support a harm reduction model; take steps to minimize tenants using drugs alone without someone aware; train staff and tenants on harm reduction specific to the PSH environment and trends/risks related to the drug supply; have and document discussions with all tenants about safer drug use and overdose risk reduction; hold events and trainings for staff and tenants focused on reducing stigma toward people who use drugs; train staff in trauma-informed care; provide additional support for tenants during higher risk periods for overdose (e.g., transitions)
Support for SUD treatment	Educate staff and tenants about different SUD treatment types, including dispelling common myths about medication to treat opioid use disorder (MOUD); regularly assess tenant SUD treatment needs and interests; establish referral pathways to MOUD providers; provide access to peer support models; provide assistance to help tenants successfully engage in SUD treatment; provide support for tenants who are already in SUD treatment or are trying to reduce their drug use and/or who are maintaining sobriety

*Legend*: Draft planned categories and examples of specific overdose prevention practices. The complete list of overdose prevention practices will be refined and finalized during the preparation phase of the study. During the 6-month intervention period, PSH buildings will be supported in implementing these practices using the strategies described in Table 1

There has been no past research that would suggest a set of “gold standard” overdose prevention practices for PSH specifically. However, the planned overdose prevention strategies outlined above have either been studied in other settings or are generally accepted as best practices, including for structurally marginalized populations such as people experiencing homelessness [38–48]. We modeled an initial list of overdose prevention practices on an overdose prevention practice package developed by an interagency workgroup for use in isolation and quarantine hotels for people experiencing homelessness during the COVID-19 pandemic. We felt this package would be promising for adaptation to PSH settings because it was comprehensive, based on expert consensus on evidence-based practices, and developed for settings bearing similarities to PSH (e.g., serving a high-risk and stigmatized population, having private rooms where overdose is possible behind closed doors). We made initial adaptations based on results from New York PSH leader surveys in the project’s exploratory phase [20], which provided insight to inform overdose prevention in PSH. For example, the surveys revealed stigma and misunderstanding related to medication for opioid use disorder, a lack of defined referral pathways for SUD treatment, and gaps in PSH staff training on naloxone use.

We are further adapting the package of overdose prevention practices for PSH settings in a 1-year preparation phase, including gathering feedback from focus groups with PSH tenants, staff, and leaders in NYC and New York’s Capital Region, as well as from the Study Advisory Board, content experts, and other relevant stakeholders. We will adapt, refine, and finalize the list of overdose prevention practices based on this feedback. We hypothesize that adapting the overdose prevention practices for the specific PSH setting will increase the likelihood that they are viewed as acceptable and appropriate by PSH staff and tenants, and that they are implemented with fidelity and sustained.

Buildings participating in the study will receive education, training, and support for implementing the overdose prevention practices during their randomly assigned 6-month intervention (technical assistance) period. While buildings will be trained on and receive support in implementing each of the overdose prevention practices, we will not require and do not anticipate that all buildings will implement every practice. The primary goal of the study is to examine how successful buildings are in implementing the practices and we expect there to be heterogeneity across buildings.

### Randomization

**Fig. 1:**
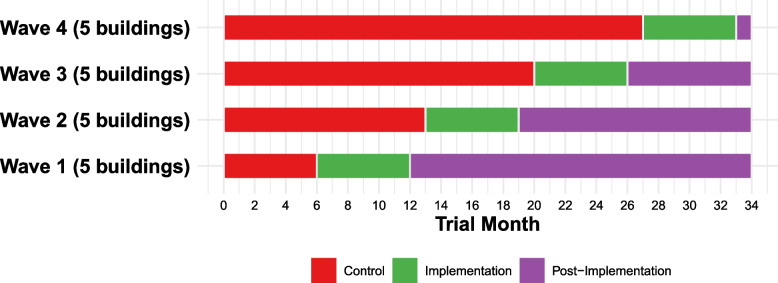
POP study stepped-wedge RCT design Legend: Schematic of the stepped-wedge RCT design. Each building will be randomized to one of 4 waves during which they will receive the 6-month technical assistance intervention. There is a 1-month break/buffer period between each wave to facilitate measurement (e.g., staff surveys and fidelity checklist completion) and preparation for the subsequent wave. Not shown in the figure is that formal measurement of sustainment will occur for each building in the 8th month following wave completion

Buildings will be randomly assigned to one of four sequential 6-month intervention waves (five buildings per wave). With the stepped-wedge design, all study buildings will begin in the control condition. Buildings are randomly assigned to cross over at different times, with all eventually receiving the intervention. See Fig. 1 for a schematic of the stepped-wedge cluster RCT design.

Randomization will be conducted by the study statistician, who will not have contact with the PSH buildings. There is no stratification of randomization. Buildings will be notified of their assigned intervention wave before the first wave starts.

### Outcomes

As a hybrid type 3 effectiveness-implementation trial, this study aims primarily to examine implementation outcomes and secondarily to explore clinical effectiveness.

#### Primary outcome

The primary outcome is building-level fidelity to the overdose prevention practices. This outcome will be measured with a scored building-level fidelity checklist completed by two raters blinded to building wave assignment. Checklists will be completed for each participating building at five time points: once before the first intervention wave begins, and once as each of the four waves ends. Raters will complete the fidelity checklists using a combination of program material review (e.g., lease language, building overdose prevention plan, training calendar and attendance logs), self-assessment surveys completed by building leaders and key staff, and environmental observation when possible/applicable (e.g., presence of naloxone in the building). For each overdose prevention practice, buildings will receive a fidelity checklist score of 0 (not implemented), 1 (partial implementation), or 2 (full implementation). Scores will be benchmarked to defined, objective, measurable criteria. The two raters will independently complete checklists, and their scores averaged. The primary outcome is the average sum score on the fidelity checklist, treated as a continuous variable. The range of possible scores will depend on the final number of overdose prevention practices (for example, if there are 15 final overdose prevention practices, fidelity checklist scores would range from 0 to 30). Secondary analyses will examine fidelity checklist sub-scores (e.g., for specific overdose prevention practice categories).

#### Secondary and exploratory outcomes

Secondary implementation and effectiveness outcomes, and exploratory effectiveness outcomes, are summarized in Tables 3 and 4. Secondary and exploratory outcomes will be examined using (1) PSH staff surveys, (2) Medicaid data analysis, and (3) PSH tenant surveys.

**Table 3:** Secondary outcomes

Outcome	Measure	Data source	Timepoint(s)
*Secondary implementation outcomes*			
Adoption	Self-reported adoption of overdose prevention practices	Building self-assessment surveys^a^	Before first intervention wave, then as each of the 4 waves ends (5 timepoints)
Acceptability	Acceptability of intervention measure [49]	Staff surveys	5 timepoints as above
Appropriateness	Intervention appropriateness measure [49]	Staff surveys	5 timepoints as above
Feasibility	Feasibility of intervention measure [49]	Staff surveys	5 timepoints as above
Organizational priority	Adapted from Klein, et al. [50]	Staff surveys	5 timepoints as above
Sustainment	Fidelity checklist (see “Primary outcome” section)	Two blinded raters	8th month after end of each intervention wave^b^
*Secondary effectiveness outcomes*			
Staff knowledge about OD risk	Brief Opioid Overdose Knowledge (BOOK) Survey [51]	Staff surveys	5 timepoints as above
Staff stigma	Perceived Stigma Toward Substance Users Scale [52]	Staff surveys	5 timepoints as above
Tenant substance use emergency department (ED) visits	Visit diagnosis ICD-10 codes including poisoning, abuse, or dependence for alcohol or any drug (AHRQ HCUP classification list) [53]. Exploratory analyses will examine visits with specific diagnosis of poisoning (OD)	Medicaid data	5 timepoints as above plus 6th timepoint 6 months later
Tenant medication for opioid use disorder (MOUD) receipt	Tenant receipt of MOUD, MOUD initiation (new pharmacy claims) and adherence [54, 55]	Medicaid data	6 timepoints as above
Tenant specialty SUD treatment	Outpatient, inpatient, detoxification	Medicaid data	6 timepoints as above

*Legend*: Table shows secondary implementation and effectiveness outcomes for the stepped-wedge cluster RCT. Secondary implementation outcomes are measured via PSH staff surveys, except as otherwise noted. Secondary effectiveness outcomes are measured via PSH staff surveys and analysis of Medicaid claims data for PSH tenants

^a^Selected building leaders and key staff (e.g., staff champions) will complete self-assessment surveys as described in the “Primary outcome” section

^b^Buildings in the final intervention wave (wave 4) will complete self-assessment surveys at one additional timepoint, to allow completion of a final fidelity checklist to assess sustainment for this wave

**Table 4:** Exploratory effectiveness outcomes from tenant surveys

Outcome	Measure
Overdose	Self-reported non-fatal overdose [56] and details about the overdose
Overdose risk behaviors	9-item instrument based on past research [57], with added item on using drugs behind a locked door
Naloxone use	Current possession, carrying, knowledge of sources, motivation to carry
Overdose risk knowledge	Brief Opioid Overdose Knowledge (BOOK) Survey [51]
Motivation for treatment/risk reduction	Motivation to initiate MOUD, obtain SUD treatment, and change overdose risk behaviors, with scales measuring importance, readiness, and confidence for making a change
Stigma	Perceived Stigma Toward Substance Users Scale [52]
SUD treatment and treatment barriers	Self-reported receipt of and barriers to SUD treatment, including MOUD [58]
PSH building services	Degree of comfort discussing substance use with building staff; self-report of building services related to substance use and overdose prevention

*Legend*: Table shows exploratory effectiveness outcomes for the stepped-wedge cluster RCT. Exploratory outcomes encompass tenant-level substance use and overdose-related measures. All are assessed via surveys administered to each tenant immediately before and 12 months following the start of their building’s intervention wave

*PSH staff surveys* will be conducted at five timepoints for staff from all participating buildings: once before the first intervention wave begins, and once as each of the four waves ends. All PSH staff with tenant-facing, supervisory, or leadership roles will be invited via e-mail to complete surveys. Invitation emails will include a hyperlink to a secure online REDCap survey. Invitations to participate in surveys will be non-coercive; it will be made clear that there will be no negative ramifications for their employment if staff decline to participate. A consent document will be included for staff to review prior to deciding whether to participate. Survey content is described in Table 3. Surveys are anticipated to take less than 20 min to complete.

*Medicaid data analysis* will use statewide Medicaid data to examine tenant-level effectiveness outcomes, as outlined in Table 3. Participating PSH buildings will provide investigators with PSH tenant identifying information. Investigators will use deterministic matching procedures to link this data with New York Medicaid data. After linkage, individual identifying information will be removed to create a de-identified Medicaid dataset for analysis; a building code will be retained and each tenant will be given a unique non-identifying study code to facilitate longitudinal analyses.

*PSH tenant surveys* will be used to examine exploratory effectiveness outcomes. These surveys will be administered twice for tenants of each participating building: immediately before that building’s intervention wave starts and approximately 12 months later. Paper surveys will be mailed to each tenant with a consent document, instructions for completion, and a self-addressed stamped return envelope. Tenants will also be offered the option to complete the survey by phone or online. Tenant surveys are anticipated to take approximately 30 min to complete. The study team will take multiple steps to increase survey participation rates, including explaining to tenants that the surveys are confidential and doing direct outreach in the PSH buildings. Survey content is described in Table 4.

Buildings participating in the study will complete agreements outlining provisions for data sharing (i.e., of staff and tenant contact information for survey distribution and tenant identifying information for linkage with Medicaid data) with the study team.

### Statistical analysis

To estimate effects of the intervention on the primary outcome, we will use a linear mixed model that incorporates both within- and between-cluster information and accounts for secular temporal trends. In particular, to assess the intervention effect, we will use a model with random site effects of the form: *Y*_it_ = μ + β_1_t + β_2_I_*it*_ + β_3_I_*it*_(*t* – s_*i*_) + b_*i*_ where *Y*_*it*_ is the fidelity checklist score in building *i* during period t, for t ∈ (0, 1, …, 4). Each period is 6 months; *t* = 0 is the first 6-month period. *I*_it_ is an indicator variable; I_*it*_ = 1 if building *i* has been assigned to implementation at period t and *I*_it_ = 0 otherwise. s is the period when implementation begins for building *i*. b_*i*_ is a random effect associated with each building *i* (the deviation of the intercept for the building from the overall intercept μ). Models will take into account a general time trend and allow for the intervention effects to grow over time following implementation. Models will be fit using the lme4 package using R software. Each hypothesis will be tested using a two-sided level of significance α = 0.05. Similar models will be used for analysis of the secondary implementation outcomes.

Implementation sustainment will be measured in the 8th month after the conclusion of each 6-month intervention wave. Paired-samples *t* tests will be used to compare each building’s fidelity scores at the end of its control period with scores during the sustainment period. For buildings assigned to early waves, there is also an opportunity to measure longer term (> 12 months) sustainment within the study measurement period.

Secondary effectiveness outcomes using Medicaid data and staff surveys will use generalized linear mixed models appropriate to each type of outcome (e.g., a Poisson model to compare visit rates). Health service use variables (e.g., ED visits) will be calculated as rates when appropriate (e.g., visits per study population per time period). For all secondary and exploratory analyses, we will examine the amount and type of missing data (e.g., missing at random or not at random) and implement appropriate statistical procedures for missing data as necessary. We will examine exploratory outcomes from tenant surveys using mixed-effects regression analysis. For the post-intervention survey time point we will conduct sub-analyses limited to tenants who have lived for at least 6 months in the building.

### Power and sample size

With 20 buildings participating in the study and 4 “steps” or waves in the stepped-wedge RCT (i.e., *n* = 100 observations over time across all buildings given 5 total time points per building), we will have at least 80% power to detect an increase of approximately two-thirds of a standard deviation (*d* = 0.69) or larger in the primary implementation outcome, a meaningful and feasible difference. This assumes a moderate correlation between scores for the same building at different time points (ICC = 0.5), reflecting stable differences among buildings. Even in the unlikely event that we do not retain 20 buildings in the study, with 16 buildings we would still have at least 80% power to detect an increase of about three-quarters of a standard deviation (*d* = 0.77) in the primary implementation outcome. To make these effect sizes more concrete, if the checklist score had a standard deviation of 8 and an average score of 15 under the control condition, the stepped-wedge design has 80% power to detect an increase of about 5 to 6 points on the checklist score (e.g., an increase from 15 to 20).

### Qualitative interviews

After the intervention is delivered, we will conduct qualitative interviews with PSH building staff, as well as tenant implementation champions, to explore multilevel factors influencing implementation, including barriers and facilitators. We will use purposeful sampling to maximize breadth and utility of information gained, interviewing individuals holding different staff roles from diverse PSH buildings participating in the study. We will also interview staff and tenant implementation champions. Interviews will use a semi-structured interview guide that will capture key EPIS framework construct domains related to success of implementation (e.g., inner context, outer context, bridging factors, innovation factors). Qualitative interviews with building leaders will additionally explore penetration of the overdose prevention practices across other buildings (i.e., those not participating in the study) operated by their agency. Interview guides will be tailored to each type of key stakeholder.

Interviews will be digitally recorded and professionally transcribed. We will perform line-by-line coding of transcripts using a list of key domains identified *a priori* based on EPIS and our study goals (deductive), but allowing new themes to emerge organically from the text (inductive) in the grounded theory tradition. We will also create templated summaries of each interview and conduct matrix analysis focused on key EPIS domains [59]. We will use Dedoose qualitative research web application to assist with thematic analysis and data organization [60].

### Payments and incentives

The Corporation for Supportive Housing is providing an honorarium of $2000 per participating PSH agency plus an additional $1000 per building participating in the study. Tenant champions will receive an honorarium totaling approximately $500. Additionally, tenants and staff will be compensated by the study team for the time they spend completing surveys and qualitative interviews.

## Discussion

We describe the protocol for a hybrid type 3 stepped-wedge RCT of overdose prevention practice implementation in PSH. Changes in the epidemiology of the overdose crisis—including widening racial and ethnic disparities—highlight the necessity of concerted efforts to reduce the disparate burden of overdose faced by structurally marginalized populations. Homelessness and housing instability are strongly associated with increased risk of overdose [5, 7, 8, 61–65]. And, while PSH is an evidence-based intervention to resolve homelessness [13, 66], additional interventions are needed to reduce tenant overdose risk.

The 20 PSH buildings participating in the trial have distinct leadership teams, are geographically disbursed across NYC and in New York’s Capital Region, and are not generally accustomed to participating in research studies. Buildings are heterogenous in size, harm reduction orientation, and existing tenant services. Though these factors increase the complexity of study administration, ultimately, we hope that they enhance the real-world applicability and generalizability of the study. The partnership of academic researchers with the Metro Team of CSH, a national nonprofit organization focused on advancing best practices in PSH, is critical for enhancing the feasibility of the study. As a trusted entity in the field, CSH is well-positioned to deliver the implementation intervention to PSH buildings participating in the study and, eventually, to disseminate the study results nationally. Project planning has also included significant involvement of people with lived experience of drug use and as PSH tenants, which we believe has further enhanced the feasibility and real-world applicability of the study. Other strengths of the trial include the multiple, robust sources of data being used to evaluate implementation and effectiveness outcomes.

To our knowledge, our study will be the first controlled trial of overdose prevention in PSH. In general, little prior overdose prevention research has focused on PSH or similar housing settings, despite their being high-risk environments for overdose and potentially promising sites for interventions [34]. The majority of related studies have been conducted in PSH and SROs in Vancouver, Canada. There, researchers have conducted qualitative interviews with PSH residents identifying overdose risks related to using drugs alone in their rooms and potential benefits of safer supply medications [28]; qualitative interviews examining a tenant-led naloxone training and distribution intervention in SROs [21]; and qualitative interviews assessing use of overdose response buttons in a women-only PSH building [27]. These studies suggested risk factors and a few potentially promising interventions to prevent overdose in PSH and SROs, yet overall there remains a large gap in the literature related to effective interventions to prevent overdose deaths in these settings.

In general, very little implementation science research has been conducted in PSH or similar housing settings [26, 67–69]. Notably, interventions for substance use disorders are still primarily delivered in healthcare or other specialized settings, despite the fact that individuals generally spend only several hours per year in healthcare settings and orders of magnitude more time in housing settings. Our study sets the stage for a new paradigm of research and strategies bringing overdose prevention to where people live and spend most of their time. Additionally, through the study we will test the application of the EPIS implementation science framework in PSH settings, shedding light on its utility in such settings and examining whether modifications should be made to maximize its applicability in future housing-based implementation efforts.

Our trial does have some limitations. First, the building-level fidelity checklist we will use to assess the primary outcome has not been previously validated. However, a similar measure has been used successfully by the study team and we have described the multiple steps we plan to take to ensure rigor of this measure, including having two blinded raters independently complete each fidelity checklist [70]. A second small limitation is that outcomes based on Medicaid data will exclude a small number of tenants (anticipated to be < 10%) who do not have Medicaid. However, using Medicaid data will allow us to feasibly assess objective outcome measures for most tenants from PSH buildings in the study. As described, we are supplementing Medicaid data analysis with tenant-collected survey questionnaires. We will attempt to maximize tenant survey participation as described earlier, but there is still the possibility of selection bias in who completes surveys and social desirability bias in responses. Finally, we are testing a package of implementation strategies; our study design will not provide experimental evidence of which of the strategies were most impactful. We will, however, explore staff and tenant champion perceptions of the specific strategies in qualitative interviews. Our priority is to test a package of implementation strategies that we believe will be effective yet which is potentially feasible for broad replication in PSH. If this research finds the package was effective, we will pursue future studies to refine the most effective combination of strategies and minimum effective dose.

In conclusion, this stepped-wedge cluster RCT will test strategies to support implementation of overdose prevention practices in PSH settings. We anticipate that the knowledge gained about implementation of overdose prevention practices in PSH will be generalizable to other types of housing settings including transitional housing provided in hotels and motels (which became more common during the COVID-19 pandemic), other types of transitional housing, SROs, and public housing or other subsidized housing buildings. Ultimately, we hope that this work will inform efforts to prevent death and suffering among the many people affected by the junction of the overdose and housing crises.

## Supplementary Information

**Additional file 1:** **Table S1.** CONSORT 2010 checklist of information to include when reporting a cluster randomised trial.

## Data Availability

Not applicable.

## Abbreviations

CSH: Corporation for Supportive Housing
EPIS: Exploration, Preparation, Implementation, Sustainment framework
MOUD: Medication for opioid use disorder (e.g., methadone, buprenorphine)
NYC: New York City
PSH: Permanent supportive housing
RCT: Randomized controlled trial
SAB: Study advisory board
SRO: Single-room occupancy
SUD: Substance use disorder
